# Diagnostic Endoscopy and Clinical Characteristics of Gastrointestinal Bleeding in Children: a 10-Year Retrospective Study

**DOI:** 10.5812/ircmj.7075

**Published:** 2013-09-05

**Authors:** Mandana Rafeey, Maryam Shoaran, Hamideh Majidy

**Affiliations:** 1 Department of Pediatrics Gastroenterology, Liver and Gastrointestinal Diseases Research Center, Tabriz University of Medical Sciences, Tabriz, IR Iran; 2 Department of Pediatrics, Children’s Medical Center, Pediatrics Center of Excellence, Tehran University of Medical Sciences, Tehran, IR Iran; 3 Department of Nursing, Children's Hospital, Tabriz University of Medical Sciences, Tabriz, IR Iran

**Keywords:** Gastrointestinal Hemorrhage, Endoscopy, Infant, Child

## Abstract

**Background:**

Upper gastrointestinal (UGI) endoscopy is a tool used frequently in the evaluation of patients presented with hematemesis.

**Objectives:**

The purpose of this study was to evaluate clinical presentations and features of UGI bleeding (UGIB) in children.

**Methods/Patients and Materials:**

A descriptive retrospective analysis was carried out on the medical records of the patients. Children and adolescents aging 0 – 18 years who were presented with UGIB were recruited in the survey over a period of 10 years (2000 to 2010) in Children's Hospital, Tabriz, Iran.

**Results:**

There were 447 patients included in this study and they were all evaluated by upper endoscopy. Clinical manifestations included hematemesis (120 cases, 26.85%), melena (60 cases, 13.42%), and hematochezia (11 cases, 2.46%). Age-related analysis showed that erosive esophagities was a more common cause of hematemesis in the younger age group (under 1 year of age) with a prevalence of 37% when compared with elder children and adolescents (40%). Peptic ulcer disease was seen in only 7.4% of infants under the age of one. Overall, Esophagitis and erosive esophagitis was the most common endoscopic finding (179, 40%) .

**Conclusions:**

There were 80 (17.90%) patients receiving endoscopic hemostatic therapy. In children with UGIB, upper endoscopy is a diagnostic procedure for the categorization of underlying causes of upper gastrointestinal bleeding in children and various endoscopic lesions may be found in a patient with the impression of UGIB.

## 1. Introduction

Upper gastrointestinal endoscopy provides useful information for the diagnosis of various causes of upper gastrointestinal bleeding (UGIB). Upper GI hemorrhage is not unusual in children and may provoke anxiety in the child and his/her parents. Mostly, the clinical course is benign and approximately 80% of the patients are presented with a self-limited course of bleeding (1). The criteria for diagnostic upper endoscopy differs between pediatric gastroenterologists. The etiology of UGIB varies throughout the world, presenting geographical alterations in common disease states (2-4).

## 2. Objectives

Considering the lack of a comprehensive study evaluating the issue of pediatric GIB in our geographical area, we aimed to evaluate the clinical presentations and endoscopic findings of UGIB in children.

## 3. Materials and Methods

In this descriptive retrospective study, we investigated the data of 447 upper endoscopies performed in children aged 0 – 18 years who had been referred to the pediatric gastroenterology department with UGIB between June 2000 and December 2010 at the Children’s Hospital in Tabriz University of medical sciences. Data such as age, sex, clinical presentation, etiologies, endoscopic and histopathologic findings for each upper endoscopy were defined. The indication for endoscopy was UGIB, but associated symptoms were also determined. Esophagogastroduodenoscopies (EGDs) performed on the same child within 1year were not included, as they considered persistence of the primary basic pathology in all cases.

Bleeding (hematemesis, melena or both), history of ingesting medications that predispose the patients to GIB, history of coagulation disorders and underlying disease were recorded.

The presence of erosive esophagitis was specified either by descriptive terms (e.g., erosion, ulcer) or according to Los Angeles classification. Gastritis is characterized by the presence of inflammatory cells in the biopsy samples taken by upper GI endoscopy. Peptic ulcer isdefined as the endoscopic view of a mucosal defect, 2 – 4 cm in diameter, with a smooth base and perpendicular borders and penetration of the muscularis mucosae and lamina propria revealed by pathology. Prolapse gastropathy is diagnosed as the invagination of a part of the gastric mucosa into the lower esophagus resulting in well demarcated hemorrhagic mucosa and bleeding revealed by upper GI endoscopy. Milk allergy is defined as clinical diagnosis of hypersensitivity or intolerance to cow's milk protein in an infant.

The prevalence rates of each of these lesions were calculated for the entire group. Esophageal, gastric and duodenal biopsy was evaluated. The presence of each finding was examined separately as outcome variables in logistic regression analyses. All patients underwent upper GI endoscopy after receiving written consent from their parents.

### 3.1. Statistical Analysis

Descriptive analyses were performed using frequency distribution, means, standard deviations, proportions, and 95% confidence intervals. Cross tab analysis was used for categorical variables and the Chi square test was conducted to test the association between independent and dependent variables. All analyses were performed using SPSS 17.0 (SPSS Inc. Chicago, IL), and the significance level was set at 0.05.

## 4. Results

Totally, 2693 children underwent EGD in 10 years, 447 (16.60%) were diagnostic procedures for GI bleeding. Demographic data of the children showed that 327 (73.15%) of cases were inpatients in the children’s hospital and the mean ± SD (median) age was 6.13 ± 3.94 years; and the male-to-female ratio was 1.57. Chief complaints included only unique signs like hematemesis in 120 patients (26.8%), melena in 60 patients (13.42%), hematochezia in 11 patients (2%), hematemesis with melena in 8 patients (1.7%), and occult blood in 35 patients (7.8%) patients, but mixed presentations were seen in the rest of patients. Abdominal pain was the most common presentation with GI bleeding in children above the age of 1 year in 7.38% of cases, followed by vomiting in 3.58% of cases. In all patients, erosive esophagitis was the most common cause of UGIB [179 (40%)], followed by gastritis in 65 (14.5%), gastric erosion in 76 (17%), peptic ulcer disease in 19 (4.25%) and esophageal varices in 32 cases (7.15%) (28 patients with cirrhosis and 4 cases of non-cirrhotic portal hypertension).

Endoscopic evidence of peptic ulcer disease was found in 4.25% (19) of cases; of which only ulcer in bulb was seen in 7 (36.8%) patients and Helicobacter pylori (H. pylori) gastritis in 19% of cases. Prolapse gastropathy syndrome was found as a source of bleeding in 4.47% of patients. Other diagnoses included Mallory–Weiss tears (2.9%), duodenitis and duodenal erosions/ulcers (7.6%). Multiple etiologies were found in 5.7% and nine cases (2%) showed negative findings. (Table 1) 

**Table 1.** Endoscopic Findings of the Patients

Endoscopic Lesion	All patients; No., (%)	< 1 year; No., (%)
**Erosive Esophagitis**	179 (40)	10 (37)
**Esophageal Varices**	32 (7.15)	2 (7.4)
**Gastritis**	65 (14.5)	7 (25.9)
**Gastric Erosion**	76 (17)	1 (3.7)
**Peptic Ulcer Disease**	19 (4.25)	2 (7.4)
**Prolapsgastropathy Syndrome**	20 (4.47)	3 (11)
**Mallory–Weiss (9.6%)**	13 (2.9)	1 (3.7)
**Duodenitis and Duodenal Ulcer**	34 (7.6)	0
**Negative Finding**	9 (2)	1 (3.7)
**Total Number**	447 (100)	27 (100)
**Multiple Etiologies**	25 ( 5.5%)	1 (3.7)

Although age-related analysis showed that erosive esophagitis (37%) was a more common cause of GI bleeding in the younger age group (< 1 year of age), gastritis also was a common finding than children and adolescents (with a prevalence of 25.9% vs. 14.5%). Peptic ulcer disease was seen in only 7.4% of infants under 1 year old. Erosive Gastritis was reported in 3.7% of patients under 1 year old and was most commonly seen in age 6 – 13 years (51% of all patients with erosive gastritis).

In our study, the bleeding from esophageal varices was seen only in two patients in the first year of life. After this age, bleeding from varices occurred in 6.7% of cases, with the highest range in children 3 – 5-year-years of age.

Use of non-steroidal anti-inflammatory drugs (NSAIDs) was reported in 9 patients (2%). No history of prolonged drug usage (antiepileptic drugs or anticoagulant) was obtained.

In the rest of patient, seventy-five patients (16.7%) had underlying diseases, including neuromuscular deficits, nephrotic syndrome, Henoch–Schonlein purpura, and cow’s milk allergy consisting of 3.5% of cases. Each of chronic anemia, portal hypertension, esophageal atresia and coagulation disorder consisted of 1.3% of all patients. Diabetes, liver transplantation, convulsion and presence of a foreign body were other underlying diseases. Thirty patients had a preceding acute viral illness history. The distribution of patients according to underlying disease and endoscopic findings are shown in Table 2, respectively. 

**Table 2.** Underlying Disease with GI Bleeding in 447 Patients

Underlying disease	Frequency (Cases)	Percent
**Nephrotic Syndrome**	4	89
**Acute Viral Illness**	30	6.7
**Neuromuscular Deficits**	4	0.89
**Chronic Anemia**	6	1.34
**Henoch Schonlein Purpura**	4	89
**Portal Hypertension**	6	1.34
**Esophagus Atresia**	6	1.34
**Coagulation Disorder**	6	1.34
**Cow Milk Allergy**	4	0.89
**Anti-Inflammatory Drugs (NSAIDs)**	9	2
**Other**	5	1.1
**Total**	84	18.79

There was a direct correlation between clinical impression about the etiology of UGIB and the underlying diseases with endoscopic and histopathologic findings in 87% and 79% of cases, respectively. The endoscopic and histopathologic findings most frequently confirmed the specific disorder diagnosed clinically before the paraclinical methods were used. No complications were reported. In 25% of patients, bleeding stopped spontaneously and in 57% bleeding was stopped by medical therapy (proton pump inhibitor or octreotide), and endoscopic therapy was used in 18% (12% sclerotherapy, 4% variceal band ligation and 2% drug injection). One patient with GI bleeding due to a foreign body died because of perforation of the esophagus and artery and lack of response to surgery. Normal histopathology was found in 14% of cases whereas 9.8% of patients had a normal endoscopy; in some cases, the histopathologic evaluation was normal despite the gross endoscopic finding of a lesion.

## 5. Discussion

The incidence of upper GI hemorrhage is not well established in children. UGIB is a concerning and occasionally life-threatening problem affecting children of all ages. In the previous years, improvements have been made in the diagnostic and therapeutic modalities. The most common causes of UGIB in children vary depending on age and geographic area.

This report is one of largest case series of endoscopy and GI bleeding in children to date in our country.

In this study, we observed several interesting results. The most surprising initial result was the high prevalence of erosive esophagitis (40%) and erosive gastritis (17%) in our study.

The prevalence of H. pylori in our sample of children is much lower than in industrialized countries. This prevalence is also lower than our previous study 8 years ago, which was reported to range between 46 and 65% (5-8). The decreased amount of H. pylori acquisition could be due to the courses of therapies that the patients have already received for their various gastrointestinal symptoms. This may be accounted for an early acquisition of the bacteria in early childhood that is decreased in our country but there is need for other epidemiologic studies. H. pylori infection was more common in native Asian (Bashkirs) children than in Russian children with peptic ulcer. Therefore, there was stated a positive association between peptic ulcer disease and ethnicity (9).

According to the findings of our study, there wouldn’t be an increased risk of gastric lesions associated with NSAIDs (2%). The present study confirms that these drugs account for only a small proportion of UGIB in our sample of children. Another study using the numbers observed and applying different hypothesis yields estimates between 1,000 and 2,000 cases of UGIB per year in France, which is an incidence in the vicinity of 1 – 2 per 10,000 children per year in 2-months to 16-year-old age group (12,000,000 in the country). (10, 11) According to that study, the fraction of cases attributable to NSAIDs would be 88% of the 41% exposed in the week before occurrence, which is 36%.

However, esophagitis and gastritis were the most common causes of hematemesis in our study, whereas esophageal varices were much less common. The bleeding from esophageal varices is rare in the first year of life. Similar to our study, another survey in France showed that 37% of children in the youngest age group (under 1 year of age) presented with gastric lesions and 52% with esophageal lesions (11). The percentage of older children presenting with these lesions was 65% and 23%, respectively. Duodenal lesions were generally rare. (11).

The etiology of UGIB in children is definitely age related. In our series, peptic ulcer was the most common etiology in infants under 1 year of age. In older age, peptic ulcer disease was seen in 47.9% of patients, which had some similarity with another survey (2). A study from Australia reported 12 cases of peptic ulcer disease in 227 (5.29%) of children who underwent endoscopy for upper GI complaints (12). The differences in frequency of peptic ulcer disease in this study and our survey may be explained by the fact that the patient population was different and that this Australian study was carried out on children with various gastrointestinal symptoms. 

Mucosal inflammation (esophagitis, gastritis, and/or duodenitis) detected by endoscopy was similar in our study (44%, 37%, 5%, respectively) to one showing the occurrence of these complications in developed countries (27.3%) and in developing countries (34.3%) (13). A study considers the presence of mucosal inflammation a questionable cause for UGIB as the findings are usually not evident for UGIB (12).

The incidence of varices (7.1%) in our population was similar to that of the Western countries (10.6%), while it was more common in the developing countries (23.4%). This finding may be related to the underlying geographic disease states resulting in UGIB. In developing countries, variceal bleeding due to extrahepatic portal venous obstruction is the most common cause, whereas peptic ulceration is rare. This may be attributed to neonatal conditions such as prematurity, omphalitis and exchange transfusion. However, variceal bleeding was among the less common underlying causes in our study (14).

There was a high correlation between the clinical impression about the etiology of UGIB and the diagnosis confirmed with endoscopic and histopathologic findings in our study. Previous study findings have yielded a very large range of results about the correlation between the clinical impression and the endoscopic and histopathologic findings. In some studies, endoscopic appearance correlates poorly with histologic diagnosis in the gastroesophageal mucosa and it is suggested that regardless of the appearance of the mucosa, routine biopsy during upper GI endoscopy should be encouraged. In other studies there is a significant correlation between these parameters. (13-16). The wide variety and controversial results in previous studies may be attributed to the differences in the study design and patient groups.

The overall yield of endoscopy in our patients is similar to that in most reports. There are several limitations to this study, including its retrospective nature and lack of adequate follow-up for some patients.

Our experience shows that upper endoscopy is a diagnostic procedure for categorization of the underlying causes of upper gastrointestinal bleeding in children, and the various endoscopic lesions that might be found in a patient with UGIB impression.
